# Widening and Elaboration of Consecutive Research into Therapeutic Antioxidant Enzyme Derivatives

**DOI:** 10.1155/2016/3075695

**Published:** 2016-04-11

**Authors:** Alexander V. Maksimenko

**Affiliations:** Institute of Experimental Cardiology, Russian Cardiology Research-and-Production Complex, Ministry of Health Care of Russian Federation, 15a 3rd Cherepkovskaya Street, Moscow 121552, Russia

## Abstract

Undiminishing actuality of enzyme modification for therapeutic purposes has been confirmed by application of modified enzymes in clinical practice and numerous research data on them. Intravenous injection of the superoxide dismutase-chondroitin sulfate-catalase (SOD-CHS-CAT) conjugate in preventive and medicative regimes in rats with endotoxin shock induced with a lipopolysaccharide bolus has demonstrated that antioxidant agents not only effectively prevent damage caused by oxidative stress (as believed previously) but also can be used for antioxidative stress therapy. The results obtained emphasize the importance of investigation into the pathogenesis of vascular damage and the role of oxidative stress in it. The effects of intravenous medicative injection of SOD-CHS-CAT in a rat model of endotoxin shock have demonstrated a variety in the activity of this conjugate in addition to prevention of NO conversion in peroxynitrite upon interaction with O_2_^∙−^ superoxide radical. Together with the literature data, these findings offer a prospect for the study of NO-independent therapeutic effects of SOD-CHS-CAT, implying the importance of a better insight into the mechanisms of the conjugate activity in modeled cardiovascular damage involving vasoactive agents other than NO.

## 1. Introduction

A considerable role of enzymes in human body has prompted an extensive research of their derivatives for medical application. The intense initial period of the research promoted the development of “new biology” [1] and biopharmacology [2]. A decrease in the number of studies of proteins for therapeutic purposes was followed by great diversity of enzyme modification methods. Some enzyme derivatives have found successful clinical application. For instance, investigation and development of plasminogen activators provided a current set of protein preparations for clinical thrombolysis [3]. Their effectiveness urges further improvements, such as a decrease in molecular mass (compared with parent molecules) or an increase in it upon construction of new modified agents [4]. Fast development of glycobiology [5, 6] leads to new approaches and targets for the future protein therapy. Nanotechnologies allowed large biological conjugates for further investigation [3, 7–9], and “smart” biopolymers responding to temperature, рН, and effects of biomolecules [10] were developed. New conceptions contributed to further diversification of protein agents, new protocols of their clinical trials, and appearance of novel aspects in the search for medical enzyme derivatives.

## 2. Improvement of Enzyme Agents by Modification

Modification of enzymes to overcome their disadvantages for medical use (low stability in the body, short retention period in the bloodstream, low resistance to inhibitors, etc.) and to add to them new properties (ability to concentrate in the damaged area, reduce toxicity, etc.) has been successfully used for a long time [3, 4]. Covalent binding of low-molecular-mass heparin to Cu, Zn-superoxide dismutase increased the enzyme stability in acid and alkaline media as well as its resistance to trypsinolysis and temperature [11]. Conjugation of bovine pancreatic ribonuclease with poly[N-(2-hydroxypropyl)methacrylamide] prolongs the enzyme retention in the circulation and increases its proteolytic stability and resistance to inhibitors [12], thus enhancing antitumor activity of the ribonuclease. Antioxidant effect of ovalbumin and antimicrobial activity of lysozyme increase after glycosylation with galactomannan [13]. Covalent binding of peptides with albumin enhances their antiviral activity and* in vivo* half-life [14]. Polyethylene glycol (PEG) is often used to prolong the effects of enzyme derivatives in the organism. After PEGylation, recombinant human hyaluronidase markedly suppresses the growth of pancreatic tumor in mice [15]. PEGylated lysozyme displays activity in a wide рН range, stability at 50°С, and high resistance to proteolysis [16]. New methods for controlled PEGylation of peptides and proteins using transglutaminase have been suggested [17]. Modification with the amphiphilic polymer poly(N-acryloylmorpholine) was used as an alternative to PEGylation [18]. Recent evidence demonstrates the diversity and effectiveness of protein modifications for the development of new medical protein derivatives [3, 4, 19, 20] and evaluates the role of protein modifications in various physiological and pathological processes [21].

## 3. Diversity and Generalization of Interdisciplinary Studies of Protein Drugs

Optimization of technologies for production of protein derivatives and their clinical application have broadened the prospects of employing proteins as potential candidates for pharmacological agents. Protein-based products for biomedical purposes (compositions, combined drugs) [22, 23] were determined, new methods of their production and isolation were suggested [24], and the concordance between structural modifications of biopharmaceutics and their therapeutic effectiveness and safety was evaluated [25]. It should be noted that development of protein drugs has numerous stages, such as choice of modification method, optimization of modification, analysis of the active agent binding and release, cytotoxicity and toxicological parameters, statistically significant confirmation of therapeutic effects* in vivo*, study of biodistribution of derivatives, and evaluation of technological approaches [4, 10]. This has provoked a vast array of publications in biochemical, physiological, clinical, and biotechnological journals, which accelerated distribution of information but hampered a presentation of consecutive evidence concerning a given pharmaceutical agent. Therefore, papers selected due to semantic search technology (for a concrete test object) were provided by electron libraries [26–28]. An analysis and review of the results obtained in interdisciplinary study of protein derivatives for medical application seems relevant in this situation. The present review deals with consecutive studies of the superoxide dismutase-chondroitin sulfate-catalase (SOD-CHS-CAT) conjugate, a supramolecular modified enzyme derivative.

## 4. Combined Effect of Antioxidant Enzyme Conjugate in Oxidative Stress

In the norm the activity of oxidation-reduction (redox) system in the body is countervailed by that of antioxidant system [29]. Shift in this balance towards overproduction of reactive oxygen species facilitates the development of oxidative stress with nonselective structural and functional damage to biological molecules and progression of pathology. Oxidative stress can be prevented and blocked by antioxidant therapeutic agents [30, 31], antioxidant enzymes among which being effective means to achieve this goal [32]. Modification of these enzymes improves their bioavailability, reduces effective dose, and increases the efficacy of therapy [4, 29, 30, 32–34].

The basis for successful antioxidant therapy is laid by the use of antioxidants at early stages of pathology, their localization and effective concentration in the damaged area, sufficient retention time in the body, safety, absence of toxic product accumulation, and beneficial interactions with body systems, for instance, immune system [29, 30, 32]. Neutralization of active oxygen species at the initial stages of their production is strongly associated with an effective blockade of damage induced by oxidative stress. This goal is achieved with help of superoxide radical scavengers at the early stages of chain transformations associated with oxidative stress. Superoxide dismutase (SOD), an enzyme that catalyzes the dismutation (or neutralization/partitioning) of the superoxide radical (*О*_2_^∙−^), can be employed as an interceptor. Hydrogen peroxide (Н_2_О_2_) which in excessive amounts contributes to oxidative damage should be detoxified with catalase (CAT). This enzyme catalyzes Н_2_О_2_ breakdown to harmless water and oxygen. Connected activities of SOD and CAT effectively block *О*_2_^∙−^ and Н_2_О_2_, when hydrogen peroxide, a product of SOD-catalyzed reaction, turns into substrate for CAT.

*Schemes  of SOD-  and  CAT-Catalyzed Reactions.* Consider the following:

(1) $$\begin{array}{c} \begin{array}{c} {{О}_{2}^{}}^{∙-}+{{О}_{2}^{}}^{∙-}+2H^{+}\overset{SOD}{\to }H_{2}O_{2}+O_{2}\\ H_{2}O_{2}+H_{2}O_{2}\overset{CAT}{\to }2H_{2}O+O_{2}\\ 4{{О}_{2}^{}}^{∙-}+4H^{+}\overset{SOD/CAT}{\to }2H_{2}O+3O_{2} \end{array} \end{array}$$

It is noteworthy that these conversions should be effectively realized in the damaged area which is often confined to the vascular wall. Antioxidant agents, including enzyme derivatives, have been used to prevent oxidative damage to blood vessels.

**Figure 1:**
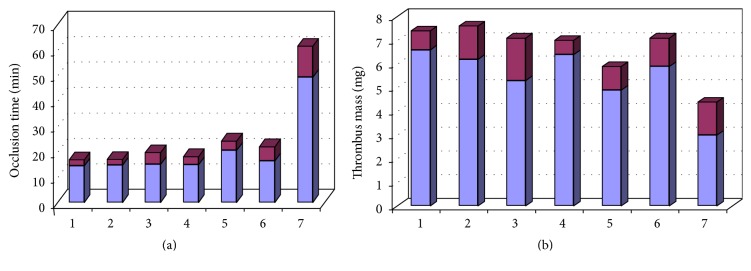
Dark areas at the top of bars demonstrate intervals of antithrombotic activities of various forms of SOD and CAT according to occlusion time (a) and thrombus mass (b). 1, control (normal saline); 2, mixture of native SOD and CAT; 3, mixture of native SOD and CAT with free CHS; 4, mixture of native SOD with CAT-CHS derivative; 5, mixture of SOD-CHS derivative with native CAT; 6, mixture of SOD-CHS and CAT-CHS derivatives; 7, SOD-CHS-CAT bienzyme derivative. Each combination of the derivatives was intravenously injected into rats with arterial thrombosis induced by ferric chlorine (saturated solution). The dose was similar to that for SOD-CHS-CAT conjugate (37 ± 3 U SOD and 80 ± 3 U CAT activities).

**Figure 2:**
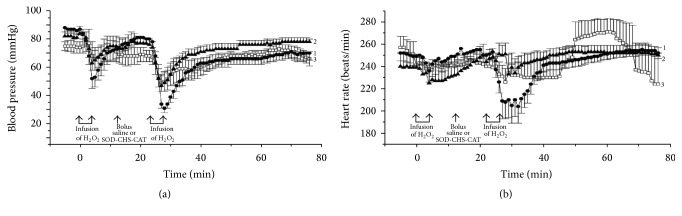
Arterial blood pressure (AP, (а)) and heart rate (HR, (b)) in anesthetized rabbits: control group (curve 1, bolus injection of normal saline), acute experiment group (curve 2, bolus injection of SOD-CHS-CAT conjugate), and prophylaxy experiment group (curve 3, injection of SOD-CHS-CAT 3 days before acute experiment with hydrogen peroxide). Arrows indicate initiation and termination of hydrogen peroxide infusion and bolus injection of normal saline or SOD-CHS-CAT conjugate (1.5 mg/kg).

We have developed a covalent water-soluble bienzyme conjugate SOD-CHS-CAT [35, 36] which offers an effective defense of the vascular wall against oxidative stress damage. Some parameters of bienzyme SOD-CHS-CAT conjugate are given in Table 1. Coupling of SOD and CAT activities in the conjugate has provided higher efficiency in comparison with individual activities of SOD and CAT and their mixtures (Figure 1) [37]. This effect emphasizes the importance of simultaneous presence of these enzymes in the damaged area (focus of injury). Enhanced accumulation of chondroitin sulfate (CHS) in vascular zones of atherosclerotic lesions has determined the choice of this glycosaminoglycan of endothelial glycocalyx for preparation of the conjugate to potentiate its vasodilatory effect [38] and rooted antioxidant activity without accumulation of concomitant toxic products [37]. Supramolecular structure of the conjugate has turned it into a nanoparticle that inhibits platelet aggregation induced by adenosine diphosphate (ADP), serotonin, or thrombin receptor agonist peptide (TRAP), which was never displayed by individual SOD and CAT [4, 39]. Preventive intravenous injection of SOD-CHS-CAT into rats 10 min before initialization of thrombosis in the carotid artery [37] or 10 min before H_2_O_2_ infusion in a rabbit or rat model of oxidative stress [39] has demonstrated a statistically significant antithrombotic effect of the bienzyme conjugate and restoration of vital hemodynamic parameters: arterial blood pressure (AP) and heart rate (HR, Figure 2). Moreover, AP and HR decrease in response to the first injection of hydrogen peroxide was negligibly small in rabbits injected with SOD-CHS-CAT 3 days before the experiment. These findings point to the importance of preventive administration of the bienzyme conjugate and are consistent with the generally accepted concept of preventive (prior to the development of oxidative damage) application of antioxidants. Efficacy of small doses of SOD-CHS-CAT in a modeled oxidative stress [4, 37] urged for further biopharmaceutical studies of the conjugate.

## 5. Block of Oxidative Stress by Preventive Administration of SOD-CHS-CAT

Preventive effect of SOD-CHS-CAT bienzyme conjugate was examined in a rat model of endotoxic shock induced by intravenous bolus injection of 15 mg/kg lipopolysaccharide (LPS) isolated from* Salmonella enterica serotype Typhimurium* with subsequent monitoring of AP, HP, and mortality rate within a given time period. The experiments were performed in cooperation with Laboratory of Experimental Myocardial Pathology, Laboratory of Stem Cells, and Laboratory of Physic-Chemical Methods at the Institute of Experimental Cardiology.

**Figure 3:**
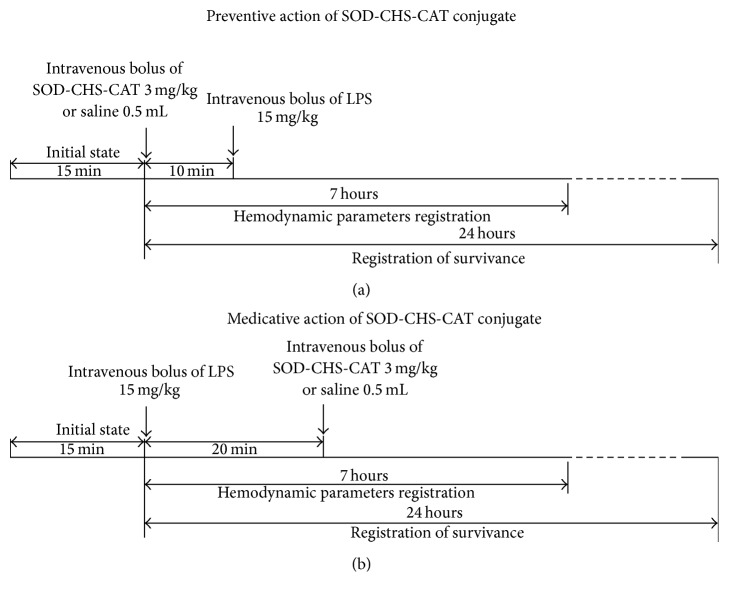
Experimental scheme with preventive (а) and medicative (b) regimens for intravenous injection of SOD-CHS-CAT bienzyme conjugate into rats.

**Figure 4:**
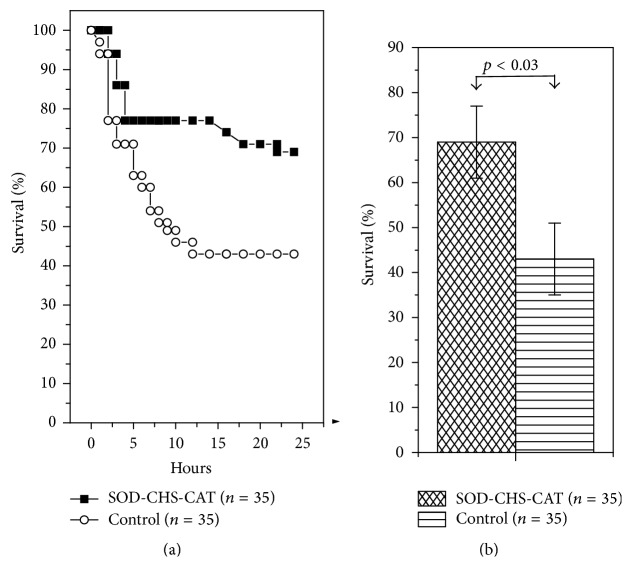
24 h lethality of rats with endotoxic shock (left, (a) Kaplan-Meier curves; right, (b) diagram presentation of the areas under these curves) after preventive intravenous injection of SOD-CHS-CAT bienzyme conjugate. Here and in other figures *n* is the number of animals in groups.

Rapid production of reactive oxygen species (ROS) in oxidative stress causes nonselective damage to biological macromolecules, thus provoking progression of pathological disorders. Administration of antioxidants before the development of damage (Figure 3(a)) is aimed at its reduction [29, 32, 40]. Injection of SOD-CHS-CAT conjugate 10 min before LPS bolus increased 24 h survival rate to 69 ± 8% versus 43 ± 8% in the control (*p* < 0.03, Figure 4). The area under the Kaplan-Meier curve in the control and experiment was 1.384 and 1.971, respectively, indicating statistically significant 1.4-fold increase in this parameter. This effect demonstrates that preventive administration of SOD-CHS-CAT increases rat viability in endotoxic shock.

## 6. Medicative Administration of SOD-CHS-CAT Is Effective against Oxidative Stress

It should be noted that endotoxin damage develops through two successive stages [40]. The first is referred to as neurological. It is associated with the nervous system reactions, develops within 20 min after LPS injection, and is blocked by injection of 2% lidocaine into the preoptic anterior hypothalamic area [41]. The second stage is referred to as cytokine-dependent, being associated with the release of the cytokines (bradykinin and TNF-*α*, interleukins IL-1*α* and IL-1*β*, and chemokines IL-6, IL-8, and IL-18) as well as increasing blood concentration of nitric oxide. It develops 20–90 min after LPS bolus injection. The absence of differences between AP and HR in control and experimental rats during the neurological stage of oxidative damage development substantiates the actuality of studying medicative activity of SOD-CHS-CAT, implying an intravenous injection of the conjugate 20 min after, but not before, LPS (Figure 3(b)), thus bypassing the neurological stage.

**Figure 5:**
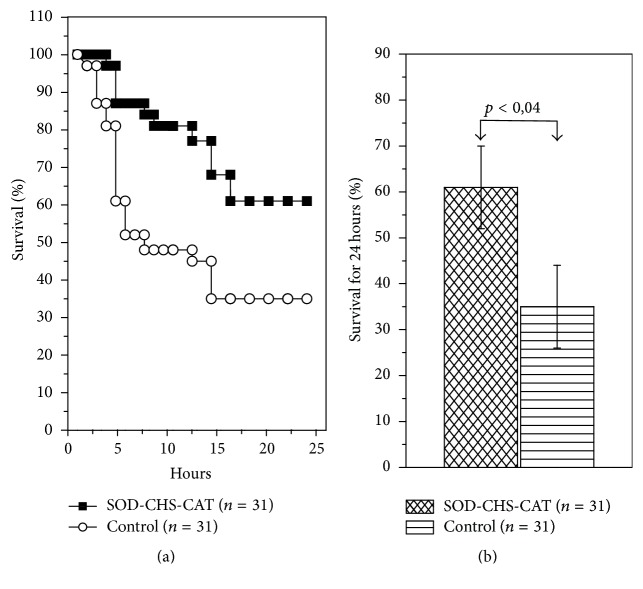
24 h lethality of rats with endotoxic shock ((а) Kaplan-Meier curves and (b) diagram presentation of areas under these curves) after medicative intravenous injection of SOD-CHS-CAT bienzyme conjugate.

After SOD-CHS-CAT injection according to the experiment scheme, survival rates of control and experimental groups were 35 ± 9% and 61 ± 9%, *p* < 0.04, respectively (Figure 5). The area under the Kaplan-Meier curve was 1.129 in control and 1.643 in experimental rats, demonstrating a 1.46-fold increase in viability.

## 7. Effects of Preventive and Medicative SOD-CHS-CAT Injection in a Rat Model of Endotoxin Shock

The similarity between AP and HR changes after preventive and medicative injections of SOD-CHS-CAT with significantly enhanced survival of rats in experimental groups demonstrates that HR increase is a compensatory reaction to AP decrease in endotoxic shock. Restoration of AP was faster in experimental rats, while intergroup changes in HR were statistically insignificant [40]. The cytokine stage of oxidative damage development was chosen as a target for successful therapeutic correction with vascular antioxidants (SOD-CHS-CAT). Effectiveness of the conjugate in preventive and medicative regimens indicated its potential wide application. Direct antioxidant effect of SOD-CHS-CAT, particularly at the initial stages of oxidative damage [39], and remote/distant therapeutic effects [23, 33] actualized elucidation of mechanisms underlying these effects. This was facilitated by changing the administration route in a rat model of endotoxin shock.

It should be noted that oxidative stress has surely and soundly become a significant and attractive target for cardiovascular prevention and therapy [42]. A decrease in oxidative stress demonstrated the efficacy of antioxidant therapy in STEMI (ST-segment-Elevation Myocardial Infarction) patients [34]. Systematic search for factors determining the success of biomedical data translation into clinical practice contributes to effective transition from the research to the clinic [43]. These trends stipulate efficacious formation of antioxidant therapy resources on the basis of biopharmacological investigations in animal [34, 42, 43].

## 8. Effect of Peroral Administration of SOD-CHS-CAT on the Development of Endotoxin Shock

Bearing in mind that peroral route of administration improves availability and spread of a drug, we designed an experimental protocol to test the effectiveness of the bienzyme conjugate administered perorally. Rats were given SOD-CHS-CAT with cottage cheese at a daily dose of 17.5 mg/kg body weight for 3 weeks (experimental group). Control rats did not receive the conjugate. The dynamics of weight gain was similar in both groups. AP, HR, and 24 h lethality were monitored after an intravenous LPS bolus injection (15 mg/kg).

**Figure 6:**
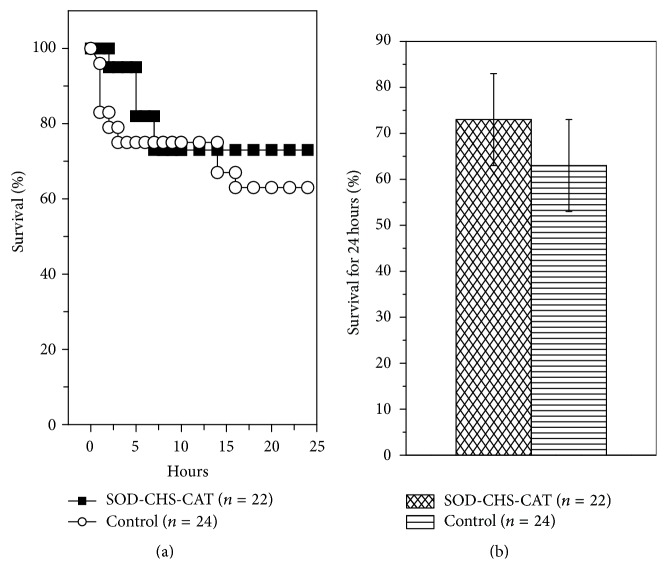
24 h lethality of rats by Kaplan-Meier curves (а) and % surviving animals by the end of the observation period (b) after intravenous injection of LPS (15 mg/kg) into rats given SOD-CHS-CAT perorally (experimental group) and rats that did not receive the conjugate (control).

**Figure 7:**
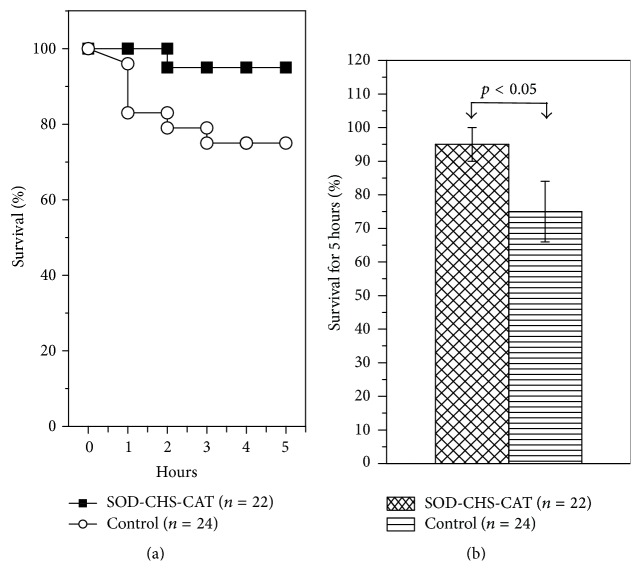
5 h lethality of rats after intravenous injection of LPS (15 mg/kg) into rats given SOD-CHS-CAT perorally (experimental group) and rats that did not receive the conjugate (control). (а) Data according to Kaplan-Meier curves, (b) according to diagram presentation of areas under the curves.

There were no statistically significant intergroup differences in survival rate: 63 ± 10% (control) and 73 ± 10% (experiment) (Figure 6). It is noteworthy that lethal outcome was earlier in control rats, as evidenced by statistically significant differences 2, 4, and 5 h after LPS injection (*p* < 0.05). During a 5 h period the areas under the Kaplan-Meier curves were 0.671 ± 0.003 (control) and 0.800 ± 0.001 (experiment), that is, 1.192-fold greater (*p* < 0.001, Figure 7). These results indicate preservation of rat viability in the given period of endotoxic shock and point to protective fast-acting of SOD-CHS-CAT. A rapid therapeutic effect of the bienzyme conjugate suggests that its doses should be varied in an attempt to increase the effectiveness of peroral administration in a complex therapy.

The absence of statistically significant intergroup differences in 24 h lethality (Figure 6) can be associated with insufficient supply of SOD-CHS-CAT and its depletion/destruction in rat organism after peroral administration. A comparative* in vitro* study of trypsinolysis resistance of SOD-CHS-CAT and individual CAT and SOD has revealed a marked decrease in the enzyme activity of these compounds after 3 h incubation with trypsin. Destruction was confirmed by electrophoresis which demonstrated a wide band of small-molecular-weight fraction in comparison with initial samples. These findings indicate that SOD-CHS-CAT administered by peroral route is prone to proteolytic destruction.

## 9. Conclusion

The results obtained confirm statistically significant effect of experimental therapy with SOD-CHS-CAT in preventive and medicative regimens and substantiate the actuality of investigating the mechanisms responsible for this effect. Collected evidence points to a direct antioxidant effect of SOD-CHS-CAT [30, 39] and a possibility of increasing the level of endogenous antioxidant biocatalysts after administration of therapeutic antioxidants [23, 33]. Moreover, it was reported that the size of myocardial infarction in rats can be reduced by NO-independent stimulation of guanylyl cyclase [44]. ROS generation was recorded at late stages of angiotensin II-induced hypertension [45] and mechanosensor regulation of angiotensin-converting enzyme was observed in response to endothelial shear stress [46, 47]. These data demonstrate NO-independent pathways for realization of vasodilatation/constriction. In addition to NO defense against conversion into peroxynitrite upon interaction with superoxide radial, the diversity of protective effects provided by antioxidants shows a prospect for a research in the mechanisms involving various vasoactive agents (not only NO) and determines the pathways for NO-dependent and NO-independent therapeutic effects of SOD-CHS-CAT bienzyme conjugate. The research into vascular damage pathogenesis and evaluation of the role of oxidative stress in it (time and place) are of paramount importance.

Production of novel protein drugs is strongly dependent on the development of biopharmaceutical industry. It has been generally accepted that producing companies should choose only two goals from the three major ones: high product quality, speedy development/production, and low cost of the product [48]. However, current situation urges the industry to achieve these three goals simultaneously. Undoubtfully, high quality, fast production, and reasonable price require advanced technologies and business strategies (the use of biomimetic metabolic pathways, breakthrough therapeutic schemes, competition in the most promising areas, etc.). It has been suggested that this will be facilitated by smaller markets and small-scale products of biopharmaceutical industry for next ten years [48]. Such a development can accelerate production of drugs based on antioxidant enzymes.

Our findings demonstrate the effectiveness of SOD-CHS-CAT conjugate in prevention and medication of oxidative stress damage, attract attention to the elucidation of mechanism of its action (probably, especially via NO-independent pathway), and emphasize the increasing actuality of the research into pathogenesis of cardiovascular disorders and contribution of oxidative stress to them.

## Figures and Tables

**Table 1:** Some characteristics of SOD-CHS-CAT derivative.

Parameters of bienzyme SOD-CHS-CAT conjugate	
Appearance of derivative	White-gray powder
Content of protein in preparation	5–7%
Specific SOD activity	43–47 U SOD/mg preparation
Specific CAT activity	70–75 U CAT/mg preparation
Effective dose for intravenous bolus administration *in vivo*	1-2 mg preparation
Recommended dose (according to enzyme activity) for acute injury *in vivo*	80–90 U SOD/kg 145–185 U CAT/kg

## Abbreviations

ADP: Adenosine diphosphate
AP: Arterial pressure
CAT: Catalase
CHS: Chondroitin sulfate
HR: Heart rate
LPS: Lipopolysaccharide
PEG: Polyethylene glycol
ROS: Reactive oxygen species
SOD: Superoxide dismutase
SOD-CHS-CAT: Superoxide dismutase-chondroitin sulfate-catalase conjugate
TRAP: Thrombin receptor agonist peptide.
